# Controlling Plateau-Rayleigh instabilities during the reorganization of silicon macropores in the *Silicon Millefeuille* process

**DOI:** 10.1038/s41598-017-07393-4

**Published:** 2017-08-03

**Authors:** M. Garín, C. Jin, D. Cardador, T. Trifonov, R. Alcubilla

**Affiliations:** 1 Grup de recerca en Micro i Nanotecnologies, Departament d’Enginyeria Electrònica, Universitat Politècnica de Catalunya, c/Jordi Girona 1–3, 08034 Barcelona, Spain; 2 Barcelona Research Center in Multiscale Science and Engineering, c/ Eduard Maristany 10–14, 08019 Barcelona, Spain

## Abstract

The reorganization through high-temperature annealing of closely-packed pore arrays can be exploited to create ultra-thin (<20 µm) monocrystalline silicon layers that can work as cheap and flexible substrates for both the electronic and the photovoltaic industries. By introducing a periodic diameter modulation along deep etched pores, many thin layers can be produced from a single substrate and in a single technological process. Besides the periodicity, the exact shape of the modulation also has a profound impact on the process and subtle profile changes can lead to important differences on the process outcome. In this paper we study both theoretically and experimentally the effect of the initial profile on the pore reorganization dynamics and the morphology of the thin layers obtained through annealing. We show that process reliability, annealing time and final layer characteristics, all can be engineered and optimized by precisely controlling the initial pore profile.

## Introduction

That a free falling stream of fluid, initially of a constant radius, is inherently unstable and that will eventually break into a series of droplets with a characteristic spacing between them is a familiar and well understood effect known as the Plateau-Rayleigh instability^1–3^. Beyond its academic purpose in fluid mechanics, this effect has demonstrated to be of great significance in many applications such as ink-jet printing^4^, microfluidic lab-on-chip systems^5, 6^, drawing of optical fibres^7^, and the long-term stability of electronic devices^8, 9^, just to name a few. Furthermore, the general underlying principles of this instability has proven to be successful in diverse areas of knowledge covering from the very small, governing the instabilities of solid nanostructures and thin films^9–12^, to the very large, having been proposed as an analogy to the Gregory-Laflamme instabilities in black strings^13^.

A particular case of Plateau-Rayleigh instabilities occurs during the high-temperature annealing of pores etched on crystalline Silicon. When the annealing is done under deoxidizing ambient the pores evolve by surface diffusion tending to reduce its surface energy, giving rise to instabilities very much like in the case of a fluid cylinder even though the process occurs in solid phase. The result is the formation of one or more bubbles beneath the surface maintaining the monocrystallinity of the surrounding Silicon. When a closely packed array of pores is annealed, the trapped bubbles can contact laterally collapsing into a spacing layer and creating a free-standing high-quality monocrystalline thin layer on top. This process, so-called *empty-space-in-silicon* (ESS), was devised by Mitsushita and Sato^14, 15^ as an advanced substrate for developing silicon-on-nothing electronic devices. Later, Depaw *et al*. proposed to use it as a kerf-less wafer slicing method, by pealing-off the free-standing monocrystalline layer, for developing ultra-thin (1 µm thick) solar cells^16, 17^.

The ESS method has been primarily used to create a single thin layer per annealing process. If additional layers are desired the remaining substrate must be polished and reused, which has serious implications in terms of cost and yield. As an alternative we recently demonstrated that deep pores with a particular in-depth pore profile can be used to slice a wafer up into multiple thin monocrystalline films in a single technological step^18, 19^. We termed this process “*Silicon Millefeuille”* since all produced layers are free standing after the annealing, which can be exfoliated into separate thin substrates. In order to produce this all-at-once result, we electrochemically etched very deep pores in the wafer with alternating narrow and wide pore diameter sections that develop during the annealing into the solid and spacing layers, respectively. Electrochemical etching of silicon^20^ is particularly well suited for this purpose as its versatility in terms of in-depth porosity control^21^ is unmatched. The distinctive *Millefeuille* profile allows defining both the number of layers and their thickness, which does not need to be the same for all produced layers. Also, the achievable thicknesses can range from a few microns, like in the ESS technique, to tenths of microns, although macro bubbles will appear inside the layer in that last case.

The flexibility offered by the in-depth pore modulation is one of the key features of the *Millefeuille* process that is yet to be fully understood and exploited. In this paper we present a comprehensive study, both theoretical and experimental, of the effect of the initial pore profile on the dynamics of the pore transformation during the annealing, and how these changes define the outcome of the process. It turns out that the in-depth profile, in addition to defining the number and thickness of the produced layers, also has a profound impact on the stability of the process, the total annealing time needed, and the final morphology of the layers in terms of bubble formation and surface roughness. Furthermore, we propose that a precisely designed profile can be used to control and engineer such layer characteristics, *i.e*. to control the number, size, and distribution of microbubbles and the surface roughness, which would have relevant consequences in their optical behaviour. For instance, especially crafted ordered distribution of bubbles within the thin films could act as an integrated light trapping mechanism optimized to boost light absorption through both scattering and resonant effects. Last, but no least, we show that a strong diameter modulation is not always desirable nor essential to produce multiple layers in a single process, opening the possibility to apply deep RIE techniques in the *Millefeuille* process.

This paper is organized as follows. First of all we will establish the theoretical background by introducing the physical model that describes the evolution of cylindrical pores during the annealing and by revising the transformation/evolution of straight pores. This example, will give us an intuitive understanding of certain collapsing dynamics that we will find later when dealing with modulated pores. Next, we will present the battery of experiments we have prepared for the study. Three different initial pore shapes have been considered namely square, saw-tooth and sinusoidal, with different in-depth modulation periods. The analysis of the resulting structures after the annealing will reveal several patterns that will constitute the focus of the paper. These patterns will be discussed under the light of numerical calculations.

## Theoretical Background

When a crystalline silicon microstructure is annealed at high temperature under appropriate deoxidizing ambient (H_2_ or Ar), it changes its shape in order to reduce its surface free energy. At temperatures below the fusion point, this process occurs in solid phase by surface diffusion, maintaining the crystalline nature of the structure. Surface diffusion is a process driven by gradients in surface curvature, *i.e*. surface atoms at high curvature regions present a higher chemical potential than atoms at low curvature regions. As a consequence, a flow of adatoms appear, from high to low curvature regions, that tends to reduce such differences. This process was studied by Mullins^22^ who showed that, for an isotropic material, the surface current of atoms, ***J***_s_, is described by

1 $${J}_{s}=\frac{-{D}_{s}\gamma {\rm{\Omega }}{\rm{v}}}{kT}{\nabla }_{s}h$$

where ∇_s_ denotes the surface gradient operator, *h* = *½(κ*_*1*_ + *κ*_*2*_ *)* is the mean curvature being *κ*_*1*_ and *κ*_*2*_ the principal curvatures, *D*_s_ is the surface self-diffusion coefficient (isotropic), γ is the surface free energy per unit area, Ω is the molecular volume and v is the number of atoms per unit area. Notice that this expression is assuming an isotropic diffusion behaviour, which is a good approximation for annealing temperatures above 1050 °C^23^. The rate of advance of a surface element in the normal direction, *dn*/*dt*, will be proportional to the divergence of the current and Ω, leading to the Mullin’s equation for surface diffusion

2 $$\frac{dn}{dt}=B{{\rm{\Delta }}}_{s}h$$

where **Δ**_s_ denotes the Laplace-Beltrami operator and *B* is a constant defined as

3 $$B=\frac{{D}_{s}{{\rm{\gamma }}{\rm{\Omega }}}^{{\rm{2}}}{\rm{v}}}{kT}.$$

The above equations are scale invariant and, therefore, it is convenient get rid of the constants by normalizing to a characteristic length *a* leading to canonical equation

4 $$\frac{dN}{d{\rm{\tau }}}={{\rm{\Delta }}}_{s}H,$$

where *N* = *n*/*a* is the normalized distance normal to the surface, *H* = *ah* is the normalized mean curvature, τ = *tB*/*a* ^4^ is the normalized time, and *a* is the characteristic length.

In order to simulate the transformation of pores under annealing, we have evolved the Mullins’ diffusion equation, Eq. (4), in time using a finite-differences time-domain (FDTD) method assuming cylindrical symmetry^24^. This assumption implies that we can simulate the evolution and collapse of a single cylindrical pore with an arbitrary profile. On the contrary, we cannot fully explore the dynamics of the layer formation where the collapse between neighbouring pores takes place, since the cylindrical symmetry of the pore surface breaks after lateral contact. In any case, as it will be shown in the discussion, through analysis of the evolution and collapse dynamics of a single pore it is possible to explain most, either quantitatively or qualitatively, of the features observed.

**Figure 1:**
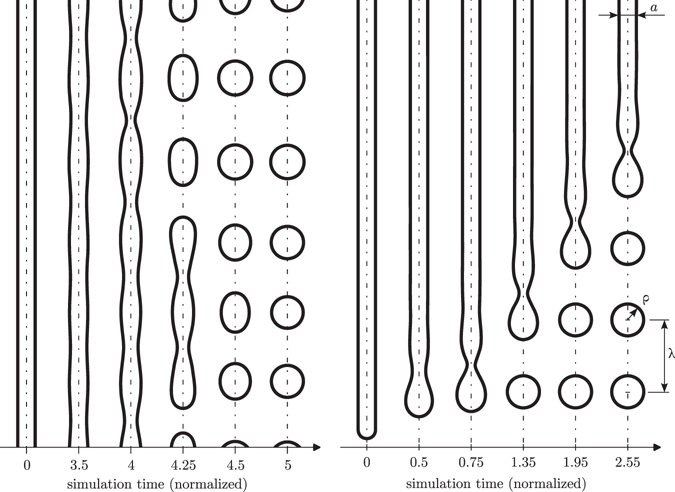
(left) Rayleigh instability in an infinite cylindrical pore. (right) Instability in a finite pore.

Before continuing, it is worth analysing what happens when a straight pore in silicon is annealed, since some of the behaviours observed in this case will be useful to qualitatively understand what is happening in certain pore profiles. An infinite cylindrical pore under the dynamics of surface diffusion is intrinsically unstable to long range perturbations following the Rayleigh instability criterion, *i.e*. it becomes unstable to perturbations with period longer than 2π*R*, where *R* is the cylinder radius. Thus, a randomly perturbed cylinder will collapse into a random string of spherical bubbles with a typical distance between them of ${\lambda }_{{\rm{0}}}={\rm{2}}\sqrt{{\rm{2}}}\pi R$, which corresponds to the fastest growing perturbation period, and a typical radius ${{\rm{\rho }}}_{{\rm{0}}}={(\frac{{\rm{3}}}{{\rm{2}}}\sqrt{{\rm{2}}}\pi )}^{\frac{{\rm{1}}}{{\rm{3}}}}R$. A similar process occurs in a finite pore etched on the surface of silicon, although in this case it is dominated by the strong perturbation that represents the ending of the pore. As result, the pore collapses sequentially from both ends into a string of spheres. This process is sometimes termed as “ovulation”, since right after the collapse the detached bubble is egg shaped, and it is much faster than the growing of random perturbations along the smooth part of the pore. This sequential ovulation leads to a string of uniform spheres as shown in Fig. 1 at the pore end. The distance between spheres is λ = 8.19 × *R*, the radius of spheres is ρ = 1.83 × *R* and the ovulation period is 0.6 time units provided that we considered *a* = 2*R*. This pattern is very precise with slight deviations at the first bubble formed at both pore ends, which depend on the exact pore start/ending shape. It is remarkable that the pore is particularly unstable at the surface and, therefore, it collapses there in less than 0.12 time units (normalized), after which it continues following the regular ovulation pattern. Also, at the centre of the pore, where both collapsing fronts meet, one can expect to find one or two bubbles that deviate from the regular pattern depending upon whether the pore can be split into an integer number of bubbles of the expected natural size.

From the above results it is reasonable to think that a closely packed array of straight deep pores could potentially be used to produce multiple layers in a single process. However, it presents several limitations that hinders its practical application, the more important being the fact that the collapsing dynamics scales with the pore radius. Any experimental variation in the pore size will directly translate into a proportional variation in the bubble size and in spacing, leading to a cumulative displacement of bubble positions along the pore axis. As a result, a slight change on the pores’ diameter over the surface, due to lithography or etching variations, will severely limit the number of layers that can be attained with reasonable yield. Another limitation of this approach is the lack of degrees of freedom to adjust the process. In fact, the only sensible parameter is the ratio between the pore spacing and the in-plane periodicity, which slightly changes the ratio between the thickness of the silicon and spacing layers produced, and that should be optimized for robustness. The practical way to change the thickness of the layers is by scaling the structure at the expense of annealing time, which grows with the fourth power of the scaling factor. For instance, to double the thickness of the final layers we should double the structure and, for the same annealing temperature, increase the annealing time by 2^4^ = 16. The pore length only allows the definition of the number of layers that would be produced.

## Experimental Details

Instead of using straight pores and relying on the ordered sequential pinching of the pores from the extremes, in the *Millefeuille* process we artificially set a diameter profile along the pore axis that defines the collapsing dynamics of the pore^18, 19^. The amplitude of the diameter modulation must be wide enough so as to prevail over both the natural instability period ${\lambda }_{{\rm{0}}}={\rm{2}}\sqrt{{\rm{2}}}\pi R$ and the sequential pinching at the pore endings. In such case, the formation of layers and spaces, both in size and number, can be fully controlled through the specific initial profile. Since the collapse of the pore occurs simultaneously all over the pore, much like in the collapse of an infinite pore due to the Rayleigh instability, there are no accumulative errors during the annealing. In other words, the variability of the fabricated porous structure does not get amplified during the annealing as it is the case in purely straight pores. Furthermore, the introduction of a diameter modulation along the pore introduces new degrees of freedom to control and fine tune the process. This pore modulation can be periodic and low-amplitude, in which case it can be analysed as a perturbation that gets amplified, or it can have a large amplitude and profit from the non-linearity of the pinching process.

In order to gain an understanding of the main effects involved during the transformation of deep modulated pores, and the formation of layers, we have realized a set of experiments with different initial profiles that shall be described first. In particular, we have electrochemically etched a total of nine silicon samples comprising the combinations of three different profile shapes and three different in-depth modulation periods. The profiles include, according to the pore radius-vs-depth function, a square profile, a saw-tooth profile, and a sinusoidal profile. The rationale behind these three different profiles is the following. The square profile, on the one hand, serves as an example of a strongly modulated pore with sharp diameter changes. The sinusoidal profile shows the diametrically opposite case, a very smoothly modulate pore. Finally, the saw-tooth profile will help us to understand the effect of introducing an asymmetry on the modulation of the pore. For each pore shape we have processed three different samples with modulation period, *L*_z_, of 8 µm, 12 µm and 16 µm, while the number of modulation cycles is 7 for all produced samples. In the plane, pores follow a perfect square lattice with an in-plane periodicity Λ = 2 µm. Besides fabrication inaccuracies, all samples have been designed targeting a minimum pore diameter *d*_min_ = 0.5 µm, a maximum pore diameter *d*_max_ = 1.5 µm and an average pore diameter of *d*_av_ = 1 µm. Finally, all samples have been annealed 3 h at 1200 °C in Argon ambient.

**Figure 2:**
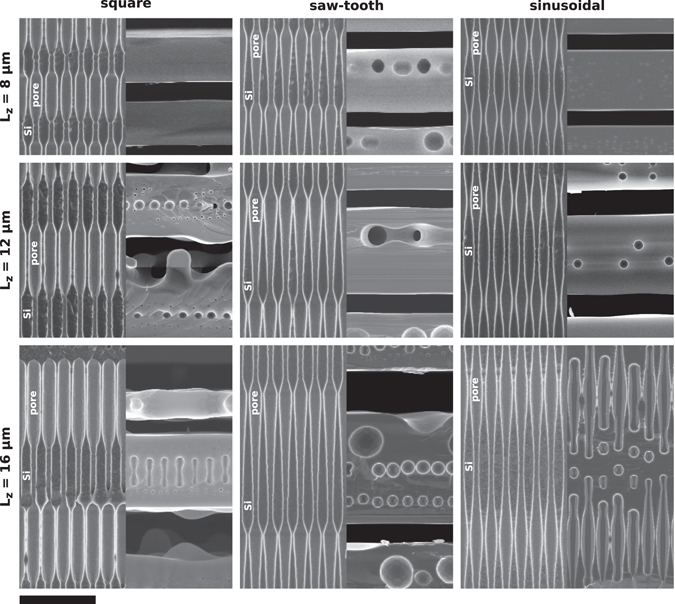
Scanning electron microscope (SEM) images, cross-section view, of samples with different pore profile before and after the high-temperature annealing. The scale bar, common to all panels, represents 10 µm.

Figure 2 shows side by side scanning electron microscope (SEM) images of the cross section, cleaved side, of all processed samples before and after the annealing. The obtained pore profiles, right after the etching, are faithful enough reproductions of the intended shapes, although the sharp diameter transitions in the square and saw-tooth profiles are limited by the fabrication method. Notice also that, in the case of the samples with square profile, the produced samples exhibit an imbalance between the narrow and wide pore sections, the former being shorter than the later, even though we initially intended both to be the same in all three periodicities. This subtle deviation will be pointed out again later, during the discussion, as it can lead to different results.

After the 3 h annealing all samples but one successfully transformed into a stack of free standing layers, several patterns emerging in the final structures that can be correlated to both the pore shape and the modulation periodicity. The results show that the square profile tends to produce silicon thin layers with a buried plane of ordered macro bubbles. These bubble planes, however, do not form for small enough modulation periodicities, while for long modulation periods it becomes a layer of elongated spheroids but, in any case, they always appear at the centre of the produced layer. On the other hand, the saw-tooth profile leads to the formation of bubbles that are not centred in the layer and that tend to be larger and partially collapsing into a percolating void layer. Furthermore, as the period becomes longer, the layer tends to trap bubbles of different sizes. Finally, the sinusoidal profile tend to produce layers with none or a few randomly distributed bubbles even though bubbles eventually appear for large *L*_*z*_ values, randomly distributed and not forming a definite bubble plane. Also, it seems that the anneal time increases as the modulation period increases for the sinusoidal profile, since the layers are not fully formed for *L*_*z*_ = 16 µm and 3 h of anneal time.

## Discussion

**Figure 3:**
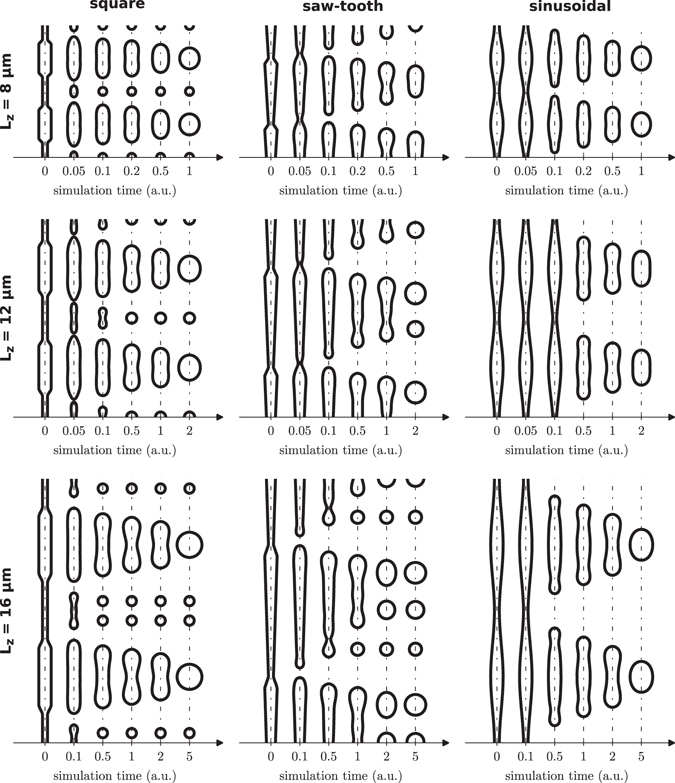
Structural evolution by surface diffusion, snapshot sequences, of infinitely-long modulated pores with different initial pore shape and different in-depth modulation periodicity, *L*_z_. For all calculations, *d*_min_ = 0.5*a* and *d*_max_ = 1.5*a*.

To shed light on the experimental results, we have first calculated the time evolution of the modulated pores subjected to transformation by surface diffusion; the simulation results are shown in Fig. 3. The basic parameters of the starting pore geometries are *d*_min_ = 0.5*a*, *d*_max_ = 1.5*a* and, in the case of the square profile, *l*_min_ = *l*_max_ = ½*L*_z_, which, fabrication inaccuracies aside, match the targeted experimental profiles with *a* = 1 µm. Evolutions are shown as a series of pore snapshots at carefully selected instants of time. Notice also that, for the sake of comparison, profiles with the same *L*_z_ value share the same timestamps so that differences in the evolution rate between them can be better observed.

In what follows we will discuss for each of the analysed profiles the main aspects related to the reorganization process, *i.e*. the pore collapse, the spacing and the surface roughness of the resulting layers. Finally we also get some insight into the annealing time needed with each profile by comparing the times needed to collapse (pinch-off) and the time needed for bubbles to contact laterally (coalescence).

### Pore collapse

#### Square profile

This is the original profile shape reported in the *Millefeuille* technique. It consists of a porous structure alternating narrow and wide pore diameter sections with an abrupt transition, which are expected to transform into the solid and spacing (empty) layers, respectively. The basic idea behind this profile was to modulate the diameter as much as possible, *i.e*. alternating between diameters close to zero to diameters close to the in-plane periodicity, so as to minimize the amount of silicon that must be moved by diffusion and, therefore, the annealing time. As shown in the simulations (Fig. 3), the narrow pore section collapses very quickly, in less than 0.05 time units with independence of *L*_z_, trapping an elongated void that eventually develops into one or more bubbles depending on the modulation length. After the collapse, the wide pore section develops in a similar fashion into a large bubble, although in a much longer time. These large elongated bubbles contract and become spherical, eventually touching and coalescing with the neighbouring ones. Since all lie in a periodic matrix along a plane, this leads to the formation of the empty layer.

The structural evolution of the square profile can be understood as the evolution of two cylinders of different diameter, which evolve at different speeds due to their different scale. First of all, the narrow sections tend to pinch-off very quickly at the ends, fostered by the abrupt diameter change at the interface between the sections, effectively separating the narrow and the wide pore sections, which further evolve and spheroidize independently. Each section will pinch-off into one or more bubbles depending on the length to diameter ratio (*l*_min_/*d*_min_ and *l*_max_/*d*_max_), larger ratios leading to pinching into a greater number of bubbles. The wide diameter section should, by design, evolve into a single large spherical bubble that would lead to the correct formation of the spacing layer by coalescence with the neighbouring ones, this normally happening before actually transforming into a perfect sphere, as already mentioned.

Due to the large *l*_min_/*d*_min_ value characteristic of this profile, the final layers obtained usually contain a perfectly 2D or 3D arrangement of bubbles symmetrical with respect the centre of the layer. In the plane these bubbles follow the initial arrangement of the pores, while in the vertical direction they are periodically spaced as dictated by the periodic pinching of a finite straight pore, *i.e*. roughly λ = 4.1 × *d*_*min*_. It must be emphasized that for a fixed *l*_min_ value, *i.e*. for a targeted layer thickness *w* ≈ *l*_min_, the trapped bubbles can be adjusted by controlling *d*_min_ and the total annealing time. On the one hand, size, spacing and number of spheres will depend on *d*_min_; for instance, a perfectly 3D cubic arrangement of bubbles can be obtained by choosing *d*_min_ = Λ/4.1. On the other hand, by precisely controlling the annealing time it is possible to frustrate the complete spheroidization of the narrow pore without impeding the formation of the spacing layer, what allows to control the shape of the trapped bubbles and produce, for instance, peanut-shaped bubbles or large cylindrical voids.

**Figure 4:**
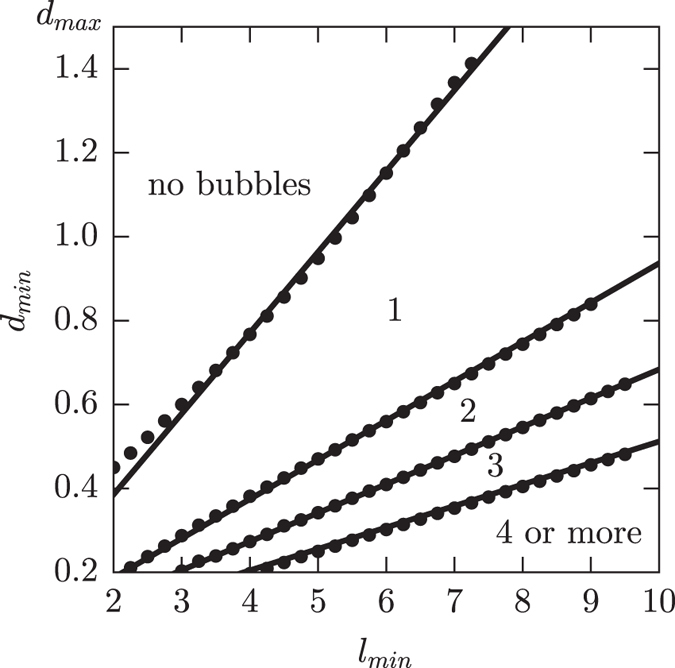
Map showing the regions where layers form with different number of bubbles trapped inside assuming *d*_max_ = 1.5*a* and *l*_max_ = *l*_min_. Threshold points were numerically calculated using an automatic search algorithm. Solid lines, crossing the origin, represent constant *l*_min_/*d*_min_ ratios fitting the points.

Depending on the application, the appearance of bubbles may or may not be desired. In the later case, bubbles can be avoided by choosing *l*_min_/*d*_min_ ratios low enough so that the closing dynamics does not allow trapping a bubble. This, can be accomplished in two ways namely reducing *l*_min_ (*i.e*. producing thinner final layers) or increasing *d*_min_. As we increase *d*_min_, however, the contrast between the two porous regions reduces and the dynamics of the pore evolution departs from the simple two independent sections. It becomes, in fact, much more similar to the evolution of a straight pore with a shallow perturbation on the diameter and, since the changes in curvature are reduced, the time required by the perturbation to grow and pinch the pore increases noticeably. Figure 4 shows a computed diagram with the *d*_min_ vs. *l*_min_ regions that lead to the formation of a layer without, or with one or more bubbles. For this computations we assumed a fix *d*_max_ = 1.5 and *l*_max_ = *l*_min_ in order to allow a straightforward comparison with the rest of the studied profiles. As the figure shows, the boundaries between the regions are nearly straight lines and, as a result, can be effectively approximated through a defined *l*_min_/*d*_min_ ratio (solid lines in the figure).

#### Saw-tooth profile

The standard square *Millefeuille* pore allows to introduce bubbles inside the layers exhibiting a mirror symmetry with respect to the centre plane of the final layers. This is, in fact, a direct consequence of the mirror symmetry that exhibits the profile itself. Further control on the bubbles forming inside the layer can be achieved by breaking this symmetry, for instance, by using a saw-tooth pore profile. This profile is composed of tapered sections with a fast transition between them, similar to the fast diameter change in the square profile. As it can be seen in Fig. 3, during the transformation this profile quickly pinches at the sudden diameter transitions and, then, each modulation period transforms into a string of spheres of decreasing size following the progressive change of the profile diameter, the total number of bubbles depending on the modulation length. Ideally, the in-plane periodicity should be adjusted so that only the bigger of these bubbles get involved in the spacing layer formation. The resulting layers produced with this profile will present a variable number of inner bubble planes, even null for small periodicities, much like the square profile but breaking the mirror symmetry. In the case of a single bubble plane, this will not be in the centre of the layer, but will be closer to one of the surfaces. Larger periodicities develop into multiple bubble planes featuring different bubble sizes as shown in the numerical calculations.

An important characteristic of the string of bubbles obtained after annealing this kind of pores is that the two largest bubbles have relatively similar sizes, what makes this profile prone to certain problems. In particular, since the pores must be close enough so that the plane with the largest bubbles can coalesce and transform into the spacing layer, this implies that the plane with second largest bubbles will be very closely packed and also close to coalescence. In the experiments it turns out that this plane of bubbles ends up partially, and randomly, coalescing and producing a percolating porous buried plane instead of a buried plane of isolated bubbles. This can be observed in Fig. 2 for *L*_z_ = 8 µm and *L*_z_ = 12 µm. It is also worth noting that the asymmetry on the transformation dynamics can also introduce an asymmetry on the surface finish of the layers. For certain modulation lengths, the expected plane of large bubbles is very close to the top surface and do not form cleanly, inducing a large surface roughness. On the other hand, this problem is not present on the bottom surface, which forms and reaches a much planar condition in the same annealing time. This can be clearly observed in Fig. 2 for *L*_z_ = 16 µm. Obviously, much longer annealing times should end up planarizing also the top rougher surface.

#### Sinusoidal profile

As we have discussed in the previous section, the square and saw-tooth profiles tend to quickly pinch-off trapping bubbles inside the final layer. Although it is possible to tweak the profile in order to minimize the formation of bubbles, it comes at the expense of either reducing the modulation length, which limits the thickness of the layer, or reducing the modulation amplitude, which makes the total annealing time larger. It turns out that a pore modulation profile that is particularly suitable for avoiding bubble trapping is the sinusoidal profile. A sinusoidal profile resembles a grown-up perturbation just before entering the non linear dynamics of the pore pinch-off. As a result, it nicely induces a transformation dynamics that lead to the formation of bubble-free layers, as calculations show in Fig. 3 for all *L*_z_ values shown. During the transformation, the profile pinches-off at the minimum radius point and, right after this moment, the pore resembles an ideally tapered cylinder, which helps avoiding the formation of small bubbles during its reorganization^24^.

Numerical calculations show that a single sinusoidal pore with *d*_min_ = .5*a* and *d*_max_ = 1.5*a* will correctly collapse into a single bubble per period for modulation lengths up to *l* = 20*a*. In principle, this would allow to easily produce bubble-free layers with thickness *w*_Si_ above 15*a* for Λ = 2*a* (*a* = 1 µm in the experiments). This is not fully consistent with the experimental results since a few bubbles start to get trapped at *L*_z_ = 12 µm and plenty of them, randomly distributed, are formed at *L*_z_ = 16 µm (see Fig. 2). There are, however, other effects that come into play at those large modulation lengths and that limit this behaviour. First of all, at large modulation lengths the pores become very smooth and the regions around the minimum diameter point starts to locally resemble a straight pore. In this situation, the roughness and imperfections of the real pores trigger Rayleigh instabilities similar to that of a straight pore, randomly trapping bubbles inside the layer, as observed in the experiments. As a result, a smooth profile such as the sinusoidal can be used to induce the formation of a pseudorandom distribution of bubbles inside the layer.

### Spacing layer and layer roughness

In the previous paragraphs we have covered the details of the pore collapsing, and bubble trapping inside the final layers. The dynamics of the bubble sintering and empty layer formation, on the other hand, also has an impact on the properties of the layers, especially on their surface. As one can see in Fig. 3, once the pore pinches off, the trapped section tends to contract and spheroidize, progressively becoming wider. When neighbouring bubbles touch laterally the pore walls dissolve introducing very high curvatures around the opening edges, triggering the process of bubble coalescence and spacing layer formation, which occurs very quickly.

**Figure 5:**
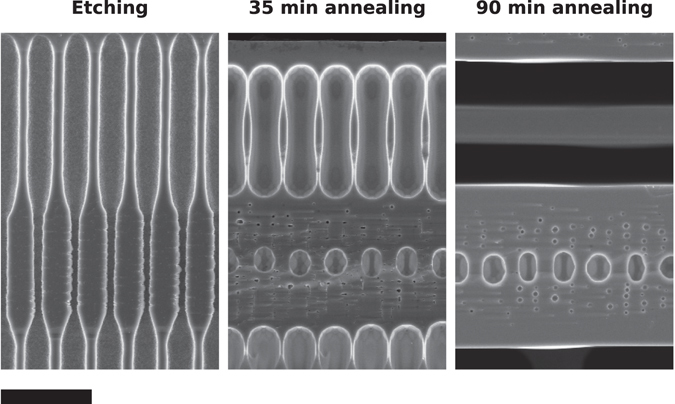
Cross-sectional view SEM images of different samples with identical starting profile after different annealing times, revealing the evolution of a structure with a large separation void which develops into a spurious layer. Scale bar is 5 µm.

Depending on the exact initial conditions (pore shape, modulation length *L*_z_, and in-plane spacing), the exact shape of the voids when the coalescence process begins can lead to different results. Let us take the square profile as an example. Right after pinching, the larger void is mostly cylindrical and its transformation into a spherical bubble can go through an intermediate peanut shape depending on the initial length of the void (*L*_max_). This intermediate peanut shape is nearly inappreciable for short *L*_max_ values (short *L*_z_), but becomes evident for larger *L*_max_ (large *L*_*z*_). See, for instance, the extreme peanut shape appearing in the square profile for *L*_z_ = 16 at one unit time in Fig. 3. As a result, two situations can be distinguished. First, the bubbles can contact laterally through the centre when they have become nearly spherical, what typically occurs for short *L*_z_ values and for optimized in-plane spacing. This is an ideal case that leads to a clean coalescence of the bubble plane leading to the formation of layers with smooth surfaces, as the experiments show for *L*_z_ = 12 µm (see Fig. 2). Second, for larger *L*_z_ values, it can happen that the voids contact laterally with a peanut shape which, in essence, means that they contact at the extremes. When this happens the dynamics of the sintering tends to become random, since it is undetermined whether the coalescence will start at the top or the bottom of the peanut, leading to the formation of rough surfaces such as it happens for in the experiments for square profile at *L*_z_ = 12 µm and *L*_z_ = 16 µm. In some particular cases, even a spurious thin layer can form in the middle of the spacing layer. Figure 5 shows in a SEM image, cross section, of a sample before, after 35 min. annealing and after 90 min annealing, in which a spurious layer forms. The image after 35 min confirms that this profile forms a peanut shape that contact at the extremes.

The above discussion explains the increased layer roughness as *L*_z_ increases that is observed in the experiments for the square profile. In fact, the layer roughness can be controlled independently of the layer thickness by appropriately choosing *L*_max_. According to the results, a smooth surface will be easily obtained for, roughly, *L*_max_ < = 5 µm, whereas a rougher surface will be obtained for larger values. Alternatively, the experiments show that the sinusoidal profile always seems to produce much smoother surfaces than the square profile. This is also explained by the particular transformation and coalescence dynamics occurring in the sinusoidal profile. As the simulations show, right after the pore pinch-off the trapped void has a particular smooth shape that progressively sharpens toward the edges. That kind of shape tends to evolve into a big bubble with minimal intermediate “peanut” shape, as opposed to the square profile. As a result, the sinusoidal profile not only prevents the bubble formation inside the layer, but also favours a correct spacing layer formation and smooth surfaces.

As opposed to the sinusoidal profile, which tends to avoid the peanut shape, the saw-tooth profile tends to favour it. This can be seen in Fig. 3 where, for *L*_z_ = 8 µm, the saw-tooth profile already forms a nice peanut bubble after 0.5 time units. However, in this case the bubble is asymmetrical and, as a result, the contact point will always be in a clearly defined plane, at the wider point of the bubble. However, during the coalescence process the high curvatures appearing on the walls tend to favour the collapsing of the structure producing a buried bubbles plane that is not predicted in the calculations shown in Fig. 3. For instance, the buried bubble layer present in the experiments for *L*_z_ = 8 µm would be explained by that fact. When this buried layer forms, both surfaces of the final layers achieve a smooth surface with the typical annealing time. For certain profile parameters, particularly as *L*_z_ increases, the buried bubble layer do not form, or form randomly, producing a highly rough surface in the opposite surface. We believe that this is what occurs in the experimental saw-tooth profile for *L*_z_ = 16 µm, resulting in an asymmetric layer with one smooth surface and another rough.

### Modulation amplitude and annealing time

In order to study the effect of the initial pore profile on the annealing time we define two characteristic times regarding the pore transformation process namely the pinch-off time and the coalescence time. The pinch-off time, *i.e*. the time needed until the pore collapses, carries information about the minimum time needed for the solid layer to form whereas the coalescence time, i.e. the time until the diameter of the pore reaches the pitch value at some point, carries information about the minimum time needed for the spacing layer to form. Since the pinch-off always happens first, the coalescence time also carries information about the minimum time needed for the whole process. The actual time needed will be, in any case, higher than the coalescence time and will vary depending on the level of smoothness desired on the surfaces but, nevertheless, the pinch-off and coalescence times offer a proper framework to compare the effect of the different parameters of the profile.

**Figure 6:**
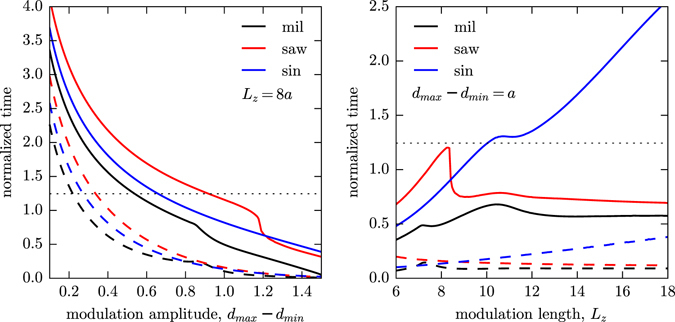
Dependence of the pinch-off (dashes) and coalescence (lines) times on the modulation amplitude, *d*_max_ − *d*_min_, (left) and on the modulation length *L*_z_ (right). In all calculations, the average diameter is *d*_av_ = ½(*d*_max_ + *d*_min_) = 1.2*a*. The dotted line represents the ovulation time for a straight pore with diameter *d*_av_.

Let us start by looking at the evolution of those times as a function of the modulation amplitude (*d*_max_ − *d*_min_) for a fixed values of the average pore diameter, *d*_av_ = 0.5 (*d*_max_ + *d*_min_) = 1.2*a*, and the pore modulation period along the *z* axis, *L*_z_ = 8*a* (see Fig. 6, left) and considering an in-plane period Λ = 2*a*. As a general rule, the evolution slows down as the profile get smoother and curvatures soften. Therefore, both times (pinch-off and coalescence) decrease as *d*_max_ − *d*_min_ increases, although the reduction of the pinch-off time is much more dramatic since the minimum diameter becomes very small for large *d*_max_ − *d*_min_ values. By the same principle, the sinusoidal profile always show the longer pinch-off and coalescence times since it exhibits the lower curvature changes for a given modulation amplitude. Conversely, the square profile show the shortest times in most of the calculated range since it presents the greatest curvature changes.

Let us now focus on the evolution of both pinch-off and coalescence times as a function of *L*_z_, shown in the right panel in Fig. 6. When the modulation length increases the only profile becoming clearly smoother is the sinusoidal and, as a consequence, the corresponding pinch-off and coalescence times increase. On the contrary, for either square and saw-tooth profiles the abrupt diameter changes are independent of *L*_z_ and, therefore, the pinch-off times remain approximately constant. With regard to the coalescence times, they also tend to reach a constant value for large *L*_*z*_ values, although it peaks at a higher time value for lower *L*_z_ values. The reason for this is that for large *L*_z_ values the coalescence occurs always through a peanut-shaped bubble, which leads to a coalescence time independent of *L*_z_. For low *L*_z_ values, however, the coalescence occurs through the equator of a spherical shaped bubble and the time increases with *L*_z_. At the transition between these two coalescence regimes, a maximum time is observed. It is worth noticing that the transition from a spherical to a peanut coalescence domain implies a reduction in time that can be rather abrupt. Also, a change in the number of trapped bubbles inside the solid layer also can lead to features in the time curves, which can lead to rather complex pinch-off and coalescence curves.

Finally it should be noted that, for periodically modulated pores, either collapse or coalescence occurs all along the pore at the same time, whereas in an initially straight pore the profile evolves starting from the extremes (Fig. 1). In fact, sequential spheroidization through the pore extremes occurs at the same time during the transformation of periodically modulated pores. Taking as a reference a straight pore with a diameter equal to the average diameter, *d*_av_ = 1.2*a*, the ovulation time would be, roughly, 1.24 time units. This time is shown as a dotted line in both panels in Fig. 6 for reference purposes. To avoid interferences from this effect, especially on the first and last layers that are closer to the pore ends, the collapse time should be smaller than the ovulation time. As it can be seen in Fig. 6, this is normally the case except for very shallow modulations. This, in effect, sets the minimum modulation amplitude that can be used in the process. Roughly, a modulation amplitude down to the range 0.22*a*–0.34*a* can be used, with the square profile exhibiting the lowest amplitude modulation. Furthermore, as the calculations show, it is reasonable to use this lower modulation amplitudes without incurring in excessively high coalescence times. For instance, in the particular case shown in Fig. 6 and for *L*_z_ = 8*a* and *d*_max_ − *d*_min_ = 0.3*a*, the coalescence time would roughly be twice the ovulation time. This might open the door to consider other etching techniques such as reactive ion etching (RIE) methods, where introducing a porosity modulation along the pore has been recently demonstrated^25^. However, further research would be still necessary to achieve a sufficient diameter modulation at the required *L*_z_. In addition, careful attention would need to be paid to the removal of the passivation layer covering the pore walls after a deep RIE etching process, which would otherwise carbonize and interfere with the surface diffussion during the annealing. This layer, a fluorocarbon polymer, usually is very inert and can be difficult to remove in some cases. Although wall smoothing with H_2_ annealing after DRIE has been successfully reported^26^ in pillar structures, the removal of the passivation polymer from very hight aspect ratio pores arrays might pose a greater challenge. Long ashing times (oxigen-fluorine plasma) and commercial solutions would probably be needed to achieve a clean silicon surface within the pores prior the annealing.

## Summary

In this paper we have studied the transformation dynamics in solid phase, driven by surface diffusion under high-temperature annealing, of ordered arrays of deep pores etched in crystalline silicon, transforming a silicon wafer into many ultra-thin (<20 µm) monocrystalline substrates in a single technological step. In particular we have shown that a precise control over the initial in-depth pore profile can be used as a powerful tool for controlling the transformation and pore-collapse dynamics and, as a result, the structural features of the produced layers such as inner porosity and surface roughness. In this work we have explored three different profiles namely square, saw-tooth and sinusoidal profiles. The square profile has proven to be able to produce layers with an ordered array (2D or 3D) of trapped macrobubbles. The saw-tooth profile, on the contrary, allows to produce ordered structures of bubbles with different sizes breaking the mirror symmetry inside the layer. Finally, the sinusoidal profile is better suited to produce thick layers without trapped bubbles, but it can be also tuned to trap bubbles with a pseudo-random distribution. The ability to control both the surface finish and the formation and distribution of macrobubbles inside the thin crystalline films could be exploited to boost light absorption without the need of resorting to expensive high-resolution lithography steps. Although further research is needed, this might lead to the production of thin crystalline substrates with good integrated light trapping capabilities for low-cost photovoltaic applications. In addition, contrary to what is referred in ref. 18, we have also shown that a strong pore modulation is not essential for layer formation although the annealing time need to be increased. This fact, however, opens the door to implement the *Millefeuille* process using DRIE techniques.

## Methods

The starting substrates are <100 > *n*-type CZ silicon wafers with both sides polished and a resistivity of 0.5 Ω·cm. First of all, a highly doped n + layer is implanted on the back of the wafers that will work both as a transparent contact and as a back surface field to minimize surface recombination losses. After the implantation, 70 nm of high-quality SiO_2_ is thermally grown while, at the same time, driving-in the implanted impurities. Then, a square array (2 µm pitch) of inverted pyramids is created on the front surface through standard photo-lithography and anisotropic wet etching. These pyramids define the sites where individual pores will grow, which would randomly nucleate otherwise. The remaining SiO_2_ is removed from the wafers with an HF solution, which are then cleaved into individual square samples of 1 × 1 inch ready for being etched. Pores are created by electrochemical etching of silicon under backside illumination in 5% HF solution. The electrolyte also contains some amount of ethanol for wetting purposes and is kept at a controlled temperature of 10 °C. In order to produce stable pores with the desired profile, applied voltage, etching current and back-side illumination all are accurately controlled throughout the process. A circular area of, approximately, 2.5 cm² is electrochemically etched in each sample.

After the electrochemical etching, samples are annealed in a horizontal quartz tube oven for 3 h at 1200 °C in a mixture of Argon with 5% Hydrogen. Samples are introduced 600 °C right after a long dip in HF (5%) to ensure that native oxide is removed from the inner pore walls. In order to keep the partial pressure of oxygen low, the gas flow is kept at 10 l/min throughout the process. Finally, samples are removed once temperature falls below 500 °C.

The structure of the samples both before and after annealing has been studied though scanning electron imaging (SEM) of a sample’s cleaved side. After the electrochemical etching, and before annealing, a small side-portion of the samples were cleaved in order to see the initial pore profile. After the annealing, the samples were cleaved again and inspected by SEM.
